# How Knowledge Is Constructed and Exchanged in Virtual Communities of Physicians: Qualitative Study of Mindlines Online

**DOI:** 10.2196/jmir.8325

**Published:** 2018-02-02

**Authors:** Sietse Wieringa, Eivind Engebretsen, Kristin Heggen, Trisha Greenhalgh

**Affiliations:** ^1^ Evidence-Based Health Care Program Department of Continuing Education University of Oxford Oxford United Kingdom; ^2^ Department of Health Sciences University of Oslo Oslo Norway; ^3^ Nuffield Department of Primary Care Health Sciences University of Oxford Oxford United Kingdom

**Keywords:** knowledge management, translational medical research, guidelines as topic, evidence-based medicine, evidence-based practice

## Abstract

**Background:**

As a response to the criticisms evidence-based practice currently faces, groups of health care researchers and guideline makers have started to call for the appraisal and inclusion of different kinds of knowledge in guideline production (other than randomized controlled trials [RCTs]) to better link with the informal knowledge used in clinical practice. In an ethnographic study, Gabbay and Le May showed that clinicians in everyday practice situations do not explicitly or consciously use guidelines. Instead, they use *mindlines*: collectively shared, mostly tacit knowledge that is shaped by many sources, including accumulated personal experiences, education (formal and informal), guidance, and the narratives about patients that are shared among colleagues. In this study on informal knowledge, we consider virtual networks of clinicians as representative of the mindlines in the wider medical community, as holders of knowledge, as well as catalysts of knowing.

**Objective:**

The aim of this study was to explore how informal knowledge and its creation in communities of clinicians can be characterized as opposed to the more structured knowledge produced in guideline development.

**Methods:**

This study included a qualitative study of postings on three large virtual networks for physicians in the United Kingdom, the Netherlands, and Norway, taking the topic of statins as a case study and covering more than 1400 posts. Data were analyzed thematically with reference to theories of collaborative knowledge construction and communities of practice.

**Results:**

The dataset showed very few postings referring to, or seeking to adhere to, explicit guidance and recommendations. Participants presented many instances of individual case narratives that highlighted quantitative test results and clinical examination findings. There was an emphasis on outliers and the material, regulatory, and practical constraints on knowledge use by clinicians. Participants conveyed not-so-explicit knowledge as tacit and practical knowledge and used a prevailing style of pragmatic reasoning focusing on what was likely to work in a particular case. Throughout the discussions, a collective conceptualization of statins was generated and reinforced in many contexts through stories, jokes, and imagery.

**Conclusions:**

Informal knowledge and knowing in clinical communities entail an inherently collective dynamic practice that includes explicit and nonexplicit components. It can be characterized as knowledge-in-context in practice, with a strong focus on casuistry. Validity of knowledge appears not to be based on criteria of consensus, coherence, or correspondence but on a more polyphonic understanding of truth. We contend that our findings give enough ground for further research on how exploring mindlines of clinicians online could help improve guideline development processes.

## Introduction

### Knowledge in Health Care, Guidelines, and Evidence-Based Medicine

The processes we use for generating, validating, and disseminating medical knowledge through clinical guidelines face growing criticism. Although many tools have been developed and implemented to support the appraisal of evidence from high-quality research studies (notably randomized controlled trials, RCTs), it is rarely, if ever, possible to fully assess and incorporate the range of evidence relevant to all the problems facing clinicians and patients in everyday practice. Knowledge from (for instance) “outbreak investigations, laboratory research, mathematical modeling, qualitative research, or quality improvement processes and clinical audit” are underrepresented in clinical guidance [1]. Furthermore, a guideline, however comprehensive, cannot address the level of granularity needed to manage the unique needs of an individual patient [2].

The mismatch between the knowledge captured in guidelines and the knowledge actually needed for clinical practice does not appear to have been anticipated by the pioneers of evidence-based medicine (EBM). They argued that clinical expertise and patient preferences should be “integrated” with best research evidence [3]. Contrary to how some critics depicted EBM *,* “best” evidence was not considered to be synonymous with a simple and restrictive hierarchy of evidence, as some clinical questions are best addressed using study designs other than RCTs or because there are some questions for RCT evidence that is impossible to obtain or unavailable [4]. Despite this early call for a pluralist approach to evidence in guideline development, standards and checklists for assessing the quality of guidelines (notably the *Grading* of  *Recommendations* Assessment, Development and Evaluation recommendations [5]) can sometimes depict an overly hierarchical approach, inadvertently privileging RCTs even when these are not appropriate and making it difficult to give appropriate weight to knowledge from non-RCT study designs.

Critics have described a number of problems with the guideline development process [6-9]. For instance, EBM’s hierarchy of evidence would promote a reductionist approach because it privileges a single epistemological position. Current guidelines may be characterized by a near-absence of heuristics [10], relying instead on complex and impracticable decision trees. There appears to be a critical mismatch between population-derived evidence and the needs of the individual patient. Shared decision-making tools and processes seem underdeveloped, making it difficult to have democratic dialogue with patients when applying guidelines in real time. Vested interests sometimes exert a distorting influence, leading to overinvestigation, overdiagnosis, and overtreatment. And single-condition guidelines appear inherently incapable of dealing effectively with multimorbidity.

Movements of scientists have recently emerged that seek to reform EBM, health care research, and guideline development [1,6,11-13] to deal with these challenges. Most notably, the Guidelines International Network has set up the AID working group [14], whose members include staff from the National Institute for Health and Care Excellence (NICE), National Healthcare Group (NHG, guideline developer for general practitioners [GPs] in the Netherlands), and comparable organizations, to identify “methods and promising initiatives for appraising and including a wider range of knowledge sources in guidelines.”

In this discussion, it is important to note that EBM and evidence-based health care (EBHC) have helped to develop numerous methods for the development and management of formal, explicit knowledge such as the structured population-intervention-comparison-outcome approach to formulating research questions, evidence hierarchies, search tools and strategies, techniques for statistical summation of trial results (meta-analysis), and so on. However, EBM scholars have largely neglected to investigate and describe how tacit, embodied, and practical knowledge is constructed in *informal* settings. This question—how is knowledge actually developed and shared in informal communities of clinicians?—formed the focus of the empirical study reported here. We’re studying GPs reflections-on-practice and knowledge-for-practice. This is one step removed from actual practice, but important in its own right.

### Mindlines

The concept of *mindlines* offers an important theoretical perspective on this question. Ethnographic research by Gabbay and Le May [15-17] demonstrated that clinicians rarely used explicit evidence from guidelines directly. Instead, they drew heavily on socially shared knowledge (which was predominantly tacit) and embodied patterns of behavior, which these authors called “mindlines.”

As a relatively new concept in the field of knowledge management, the definition of mindlines remains somewhat fuzzy. Although the term *mindlines* stuck as a suitable antonym of *guidelines*, the authors who originally coined the term are the first to admit that they are not very content with the term “lines.” This somewhat echoes Tim Ingold’s observation that “the straight line has become an icon of modernity. It offers reason, certainty, authority, a sense of direction. Too often in the twentieth century, however, reason has been shown to work in profoundly irrational ways, certainties have bred fractious conflict, authority has been revealed as the mask of intolerance and oppression, and directions have been confounded in a maze of dead ends [18].”

In their book [15], Gabbay and Le May write that mindlines should not be seen as a measurable, closed system but instead quote a GP who finds that it is “[...] more like diffuse, often bending sets of influences which vary on different days in their impact depending on what else is going on and which sometimes go in different directions.” The authors have recently presented mindlines as processes, not entities per se [19].

Many researchers have interpreted the concept in different ways, as shown in our review on the concept of mindlines 10 years after its conception [20]. This may partly be because the notion of mindlines fuses a raft of theories into one single concept of knowledge, knowledge creation, and knowledge diffusion. Notwithstanding the residual uncertainty and disagreement about the meaning of the term, however, we think the concept of mindlines is helpful in any effort to describe and understand knowledge and knowledge processes on informal networks of clinicians.

First, mindlines convey the idea of knowledge as both individual and collective. This links theoretically and empirically with the literature on communities of practice [21-23]. This concept emphasizes not only a common topic of interest but also a common (and often emotionally laden) group identity that is shaped and reinforced through group interaction. For example, in an early article on communities of practice, Jean Lave [21] exhorts those who study knowledge and its acquisition to:

...consider learning not as a process of socially shared cognition that results in the end in the internalization of knowledge by individuals, but as a process of becoming a member of a sustained community of practice. Developing an identity as a member of a community and becoming knowledgeably skilful are part of the same process, with the former motivating, shaping, and giving meaning to the latter, which it subsumes.

Second, mindlines represent both explicit and not-so-explicit knowledge. Tacit knowledge [24,25] and practical knowledge [26] form an important aspect of mindlines. They are more complex than simple cognitive shortcuts, heuristics, or rules of thumb [16]. Gabbay and Le May also refer to the socialization, externalization, combination, and internationalization processes of tacit knowledge creation [27] to explain how mindlines form.

Third, mindlines can be characterized as a form of “knowledge-in context-in practice” [17]. They consist and are shaped by many influences, including personal experience, training, interactions with colleagues, patients and industry representatives, as well as local circumstances and contextual constraints. It pushes the discussion away from barriers to knowledge translation [28] and toward a focus on the dynamic trade-offs that practitioners have to make [29].

Fourth, storytelling and casuistry are important cornerstones of the idea of mindlines. This reflects theories of narratives shared among clinicians in face-to-face interactions [30,31] and casuistry [32].

Fifth, Gabbay and Le May stress the importance of making sure mindlines “are based on the research evidence wherever possible” [16], yet mindlines appear to lack a consistent theory of validity of knowledge. This noticeable lacuna calls for further research.

By exploring the idea of mindlines theoretically and empirically in guideline development organizations and the wider community of clinicians, we hope to find innovative ways to appraise and incorporate a wider range of evidence into guidelines and also explore how guidelines are interpreted and applied in real time. In this way, we hope help to ensure that guidelines are better able to interface with the mindlines that emerge informally among communities of clinicians and the implementation of evidence-based decision making in everyday practice.

### Virtual Networks as Artifacts

New technologies (specifically, social networks, online bulletin boards, and email lists) offer new possibilities for collaborative knowledge creation that are not built into the traditional EBM framework.

We sought to explore these possibilities by examining virtual networks of physicians as part of a larger research project that aims to inform closer links between the development and use of clinical guidelines and the mindlines that emerge informally among communities of clinicians. The empirical work reported in this paper focuses on three virtual social networks of physicians in different countries. Our objectives were to describe the form and nature of knowledge, and the practices involved in knowing, of these practitioner communities; identify how mindlines develop in such communities; and explore how a broader set of knowledge sources influence (or why they fail to influence) the clinical community.

Gabbay and Le May’s theoretical work on mindlines was derived from an extensive review of the philosophy of knowledge [15], as well as extensive ethnographic research comprising direct observation of clinical practice and face-to-face discussions (eg, among local peers in practice meetings). Our own empirical work, undertaken at a time when clinicians’ peer interactions increasingly occur virtually, asynchronously and in large online communities [33-35], sought to complement the original approach taken by Gabbay and Le May.

As informal communities of exchange between GPs, Web-based networks are not unique. Informal communities of doctors in hospitals are explored extensively, for instance, in the study by Haldar et al [36]. But Web-based communities have some specific characteristics that make them particularly interesting in our context.

First, the dialogue is not limited in time and space (eg, not limited to a particular medical institution where doctors meet). Second, the access is less regulated than many other comparable arenas. There is some kind of selection (you must be a doctor, you must have sufficient electronical skills, etc), but the selection is less formal and less strict than in many other settings where doctors interact and exchange knowledge. Third, as private communities of clinicians, they provide an environment to freely discuss topics without the presence of patients or representatives of the pharmaceutical industry. Fourth, there is no formal censorship of the dialogue. The most important censorship is the *governmentality* or self-regulation of the group (eg, members don’t want to ask “stupid” questions). Fifth, they offer an efficient way of researching collective knowledge involving thousands of GPs from hundreds of sites and practices.

Sixth, it has been shown that more formal online communities, including social media, have the potential to empower health care professionals and patients to apply knowledge by involving them in the intermediation and development of that knowledge [37,38]. Some virtual communities are already successfully used to create and share new knowledge using a strict structured format of questions and replies. For example, the network sermo.com that originated in the United States and claims to have 600,000 clinicians participating, lets its members post and answer multiple choice questions, with some additional features such as adding new answers to those questions to choose from and masking other member’s answers for a few weeks [39].

This ethnographic study of online professional communities is aimed to reveal how informal collective knowledge can be built and shared. Although practice is not studied directly (hence, it is not possible to document how tacit, embodied knowledge influences specific instances of clinical practice), this methodology reveals for the first time how “mindlines“ may develop dynamically through multiple contributions to topic-based threads.

We take virtual networks to be “knowledge artifacts,” not so much as carriers of objective knowledge, but as in the words of Cabitza et al “collaboratively created [inscribed artifacts], maintained and used to support knowledge-oriented social processes (among which knowledge creation and exploitation, collaborative problem solving and decision making) within or across communities of practice” [40,41]. As such, virtual networks and mindlines share the inherent duality of knowledge artifacts as holders or as catalysts of knowledge at the same time.

## Methods

### Design

The study was set up as a digital ethnography of the interactions in virtual social network groups of physicians looking at how knowledge is formed and shaped in the online environment.

Digital ethnography takes many forms including virtual ethnography, Internet ethnography, sensory ethnography, and hypermedia ethnography, each with a slight difference in application and epistemic assumptions, but akin in their use of digital technologies [42]. These technologies, such as online questionnaires, digital video, social networking websites, and blogs, offer social scientists a multitude of new tools to do research with [43].

Digital ethnography can examine the same concepts that researchers in the humanities find useful in any kind of ethnographic research: experiences (affective, sensory, and embodied), practices (what people do), personal relationships, social worlds (as a theory of communities), things (and how they are made meaningful), localities, and events; albeit somewhat differently [44]. For instance, whereas conventional ethnography can explore the influence of physical environments on human experiences and action, digital ethnography can do the same for the digital environment.

Pink et al state five core principles of digital ethnography: there is more than one way to engage with the digital, for example, broadband, smartphones, and games (multiplicity); digital media are part of other, nondigital relationships and activities (nondigital-centricness); it is a flexible research design that can be fitted to the specific research question and context (openness); it continuously asks itself how it produces knowledge in a digital world (reflexivity); and it engages in alternative forms of communicating “beyond the standard written production of academic scholarship,” such as ongoing collaboration and dialogue with the research participants (unorthodoxy) [44].

### Networks

We had access to three social networks that provided contrasting but comparable datasets: a Facebook group of UK-based clinicians called “Tiko’s GP Group (TGG),” a virtual network set up by the professional GP societies in the Netherlands called “HAWeb,” and a network of physicians in Norway called “Eyr.” All networks have a dedicated administrator or administrative team moderating membership and online activities. TGG and HAWeb networks are closed; they can be accessed only after specific approval of a group administrator who confirms that the participant is a clinician (TGG) or a member of a medical society (HAWeb). Eyr is not closed but accessible for anyone after registering; however, in practice, almost all members are medical doctors.

The four authors of this paper are a Dutch GP who practices in both the United Kingdom and the Netherlands, a UK GP (TG), a Norwegian social scientist (EE), and a Norwegian nurse (KH). We were helped by research assistant Kristiane M Hansson (KMH).

### Data Collection

SW had already become a member of TGG and HAWeb. He had helped to develop HAWeb for the Dutch GP associations NHG and Landelijke Huisartsen Vereniging, LHV in the Netherlands after seeing and signing up for similar network groups online. In the United Kingdom, he heard from young colleagues about TGG on Facebook and signed up to get GP relevant updates and news. SW and KMH signed up for Eyr specifically for this research project. We contacted the administrator of each network to explain the project and ask for consent. We did not participate in any group discussions except the one to highlight the nature of the research and give participants the opportunity for feedback.

On the basis of the large number of posts on these virtual networks and to allow comparison across networks, we chose to restrict the focus of our study to postings relating to raised levels of cholesterol (detected by a blood test and viewed as a risk factor for cardiovascular disease) and statins (cholesterol lowering drugs such as simvastatin, atorvastatin, and related molecules). For years, statins have had a prominent position in debates in medical communities across many countries. Questions asked about statins include which subgroups of people with raised cholesterol levels will gain a significant benefit from taking a statin [45] and how to deal with patients who cannot tolerate statins—for example, because of the common side effect of muscle aches [46]. However, the prevailing debate in the medical literature concerns the interpretation of evidence on whether the benefits and harms of statin therapy have been over- or underestimated [47-49]. Some scholars argue that almost everyone should take a statin as it may prolong their life, and side effects are rare; others for whom side effects are much commoner than the results of RCTs suggest and people with minimally raised cholesterol and no other risk factors would be better to avoid these drugs. As this debate is heated and still ongoing, statins form a rich and interesting subject to explore mindlines online.

To obtain ethical approval for online research, we sought consent from the social network administrators first and then presented the research plan in an online discussion post in the networks, asking members for approval and feedback. This research was approved for each network in each country separately: by Ethics of Research Committee, QMERC at the Queen Mary University London in the United Kingdom under reference number 2014/82, by Norwegian Centre of Research Data, NSD at the University of Oslo in Norway under reference number 48032, and by Research Ethics Committee, REC at the Radboud University Nijmegen Medical Centre in the Netherlands under reference number 2016-2680. To maintain anonymity of participants, quotes in this publication have been translated from Dutch and Norwegian or paraphrased from English posts to avoid connecting them to members using the virtual networks’ native search engines.

Using the native search engines in these networks, we looked for online posts from 2013 to 2015 that contained (parts of) the following words: statins or statin, cholesterol, cholesterol-lowering treatment, cholesterol-lowering drugs, hypercholesterolemia, familial hypercholesterolemia, HMG-CoA reductase inhibitors, and their Norwegian and Dutch equivalents.

### Data Analysis

As our analysis was focused on the generation and application of mindlines in online conversations between doctors, we needed an analytical approach that reached beyond mere thematic descriptions. We therefore used an iterative part thematic descriptive and part interpretative approach [50] to analyze and synthesize the data with mindlines as a reference model. First, we were interested in themes and topics related to the participants’ use of knowledge and knowledge sources such as references to guidelines, research-based evidence, personal experiences, reported patient experiences with statins, and so on. Second, we wanted to capture the participants’ “acts of knowing” in terms of the logics underpinning their reasoning and the interactions in the online communities. We were interested not only in *what* sources they built their arguments on but *how* they built and exchanged their arguments. How did the participants construct their arguments? What characterized their “styles of reasoning” [51,52]? How did they address and invite each other into discussions? How were patient problems articulated? and what sorts of responses were given by whom?

The empirical data from online discussions were subject to preliminary analysis by each researcher independently with the aim of identifying and classifying both the aforementioned aspects of the knowledge creation in the conversations. Second, we discussed our findings as a group and compared and weighed our different descriptions against each other and against Gabbay and Le May’s description of mindlines [16]. A draft of the descriptive research results was fed back to the online communities for review and to request input for topics for discussion. Finally, in an interpretive synthesis process, the topics discussed by the online communities, key findings, and limitations of the study were drawn out through reflection and discussion among team members.

## Results

### Three Virtual Networks

Each network agreed to grant access to the research team. The TGG group of UK GPs consisted of approximately 2900 members. The HAweb group consisted of more than 30,000 members, and Eyr had 2300 to 2400 members. The activity (rate of posts) of members varied hugely both within and between networks. We found the TGG group in the United Kingdom to be by far the most active of the three. On the topic of cholesterol and statins, there were more than 1300 posts over 2 years. Most members did not contribute; all discussions were started by 64 individual members, and all posts were written by about 150 individual members. These posts varied considerably in length. Most were informal and very short, consisting of a word, an exclamation, or a link, though there were interspersed with longer postings that included segments of patient narrative or presentation of an argument. Most discussions had responses, with some threads extending beyond 30 posts. In Eyr and HAweb, there were fewer than 100 posts in 35 discussions over the same time frame. These posts tended to be longer and more formal and attracted none or a small number of responses.

TGG group was not only accessible via a browser but also via the Facebook app on a mobile phone, which made it easily accessible and integrated with other activities and friends on Facebook. Eyr and HAWeb are more dedicated services without a mobile phone app. A member had to log in first to see discussion, but would get further updates and could reply via email directly once they had engaged in one.

Many of the GPs who did post on TGG appeared to be relatively young and inexperienced (judging by their profile pictures and postings such as worrying that they may have posed a “silly question” and saying that they were doing locum work). The members on Eyr and HAWeb appeared to be more senior, as their postings featured concerns about issues relevant for practice owners or revealed extensive previous experiences relating to the issue at hand.

All networks had administrators and a protocol on rules and what kinds of postings are permitted, but in practice, moderation appeared to be very limited on these networks. No clear signs of interventions by an administrator were found on any of the threads studied (though we did not find any breaches of protocol either).

### The Shape and Nature of Knowledge Online

#### Guidelines Mentioned Only Rarely

As Gabbay and Le May’s also observed, we found few occasions where GPs referred to evidence and guidance on the TGG group. In a sample of over 1300 posts, there were just 45 links from members to online content elsewhere, 17 posts containing the word guidance, 50 with the word guideline(s), 25 with the word evidence, and 29 references to NICE (the national guideline developing organization in the United Kingdom). Of 37 links to other websites; four were to NICE, three to GP-Update (provider of specialist courses and online learning for GPs), and four to GP Notebook (a database of clinical medicine topics for GPs with a search facility). In 46 of the 76 discussion threads, the words NICE, evidence, study, guidance, or guideline were not mentioned in any post. On Eyr, members’ references to guidance or guidelines were similarly sparse, but linking to other papers and sources of information occurred more frequently: 47 times in 26 discussions. On HAWeb, there were few links to other websites, but referencing to guidelines happened in 18 out of the 56 posts over 9 discussions.

The higher proportion of posts that referred to guidelines on HAWeb and Eyr appeared to be related to the more formal nature of the posts. Many of these specific references were presented to the community as useful to solve the problem at hand and could be posted without any further commentary. Other links were posted to draw attention to medical news in the media: to the debate on the usefulness of statins in lowering cardiovascular risk, the influence of vested interests on guidelines, or how statins were discussed in new journal articles.

Strikingly, in many cases on TGG and Eyr, members did not state which guidelines or evidence they were referring to. Rather, a *nominal* perspective toward guidelines was taken (“guidelines” referred to in name only, and in a generic sense):

As the guidelines state—those suspected of familial hypercholesterolaemia should be referred to a specialist for most likely high-dose statins.

Post on TGG

I keep getting into patients in nursing homes in the age group 90+ with 80 mg statins in addition to 15 other medications. Separately certainly correct according to guidelines (written by people who are sponsored by industry).

Post on Eyr

On HAWeb, this was not the case, as GPs used the term “standard,” which is the specific name for guidelines produced by NHG (hence, from a GP-funded agency). In the Netherlands, there is almost no competing guidance for GPs, which probably explains why these GPs talked about “ *the* guideline” rather than “guidelines in general.”

#### Case-Based Reasoning

The concept of mindlines envisages knowledge being constructed collaboratively by sharing cases about patients and their situations. In the TGG group, there was a strong tendency to start a discussion or post a question based on a clinical case. In 44 out of the 76 discussion threads, initiators started off with describing a patient they had recently encountered. Statins were not necessarily the main topic on which they sought peer advice, but were mentioned as part of explaining the clinical situation. Group moderators and the GPs themselves made great efforts to protect patient confidentiality. In all cases, personal details were anonymized and adjusted to make reidentification impossible.

There were some references to actual cases in Norwegian Eyr too, but none in the Dutch network. It is not clear what explains these differences, but it may be coincidental and related to the small number of posts about statins and cholesterol, as on other topics in HAWeb members do present patient cases. An alternative explanation is that the prevailing debates among epidemiologists have taken place predominantly in the United Kingdom and attracted extensive coverage in the medical and lay press there; some exchanges have been acrimonious and linked to allegations of conflicts of interest and the threat of legal action against the *British Medical Journal*. Dutch GPs, in contrast, were exposed over the same time frame to a much more consistent message from a single, uncontested national guideline.

#### A Focus on “What” Rather Than “Why”

Where clinical cases were shared in the online group, they were mostly presented in a short, clipped, telegraphic style of writing. Elaborate narratives taking a more holistic view of the patient’s context, preferences, or social relationships were uncommon. Rather, clinical findings and test results were used to describe patients and frame the problem:

Presenting a woman 51 years. BMI around 28. Little exercise. Former smoker but stopped 5 years ago. Before this 20 pack years. BP average 140/100. Total cholesterol 6.8, LDL 4.1. Other normal. No familial risk of cardiovascular disease.

Post on Eyr

The above quote is typical of the style in which a patient was presented “objectively” to peers in the virtual network. There is a strong emphasis on quantitative biomarkers and on aspects of lifestyle such as smoking and exercise that have been identified in research studies (and the guidelines that draw on them) as risk factors for cardiovascular outcomes. In this way, the account is strikingly parsimonious, omitting aspects of the narrative that are not directly related to cardiovascular risk factors. There is no detail, for example, about how she articulates *why* she is not exercising (or what could be done about it), what made her stop smoking, her life story, family circumstances, or the way she reasons about her own health and lifestyle. Perhaps the reason for this is that the virtual forum is being used to help *interpret* the guidelines on a case-by-case basis; although other factors (such as patient preference and circumstances) will also inform the clinical decision, this is not the aspect of practice for which the posting GP is seeking input from others.

#### Emphasis on Outliers

Another finding when considering the presented clinical cases was that these posts were most frequently about unusual situations and outliers. For instance, out of the 44 clinical cases in the TGG group, 16 covered a case of familial hypercholesterolemia, a condition affecting only 0.2% of the UK population [53]. On these networks, it appears that what becomes explicit, what gets discussed, and forms the basis of learning does not reflect the bread-and-butter knowledge of clinical practice but uses unusual cases to extend and challenge that knowledge (see Discussion).

#### Material, Regulatory, and Practical Constraints on Knowledge Use

In all three networks, members frequently referred to regulations, directives, systems, and financial restrictions in which decisions were situated. They discussed knowledge in relation to their role in the welfare state, the perceived influence of industry, and important socioeconomic and ethical considerations. Recommendations on statins often conflicted with economic reality—for instance, in this post on problems relating to funding and quality indicators for statins:

Module 2013 states at indicator 11B: preferred means simvastatin alone. So no pravastatin, as is preferred in accordance with the revised standard CVRM 2012 simvastatin. But now we had run into the problem that in practice many patients in the past had side-effects of simvastatin and quit. With provide an inexpensive alternative—restarting pravastatin—you score worse on the new indicator 11B in 2013.

Post on HAWeb

Here, a GP laments that the indicator 11B recommends that he prescribe simvastatin to lower cholesterol as advised by the national guidelines on cardiovascular risk management. But if he wants to offer patients who have side effects a better alternative in the form of another cholesterol lowering drug (pravastatin), his “quality score” (according a prescription module from a health insurance company for the GP practice to gain the status of a “plus practice”) goes down. This illustrates how knowledge, and decisions based on that knowledge, are embedded in the local context in practice—and also how they are influenced by the “action at a distance” of well-intentioned national policies to improve prescribing quality but which are insensitive to the granularity of individual cases. In this case, a financial remuneration system competes with knowledge from guidelines and also goes against a solution that would seem more beneficial to the particular patient being considered in the here and now.

#### Not-So-Explicit Knowledge

The concept of mindlines entails that much of the knowledge shared is tacit, inexplicit, and more than can be told [25]. Though the very nature of tacit knowledge makes several authors suggest that this is inherently uncodifiable [54], others note that tacit knowledge can be surfaced and measured, albeit indirectly [24]. If defined as the knowledge subconsciously needed to perform things that are the focus of our attention [55], tacit knowledge can be surfaced through storytelling, modeling, shared practice, and other social interaction. On the basis of this latter understanding, there is a degree of shared knowledge assumed in most posts in our dataset. GPs would causally use concise reasoning in short sentences, which the poster knew did not need to be articulated explicitly. For example, one GP presented a case as follows:

Need LDL before thinking familial HC, def do TFT and Dm check, poss NAFLD given results, think what you done correct, I would just wait re clinic refer before getting results back in case TFT/DM show cause.

Post on TGG

This goes beyond jargon as an economic means of using language. The phrase “Poss NAFLD given results” assumes not merely that list members will know that NAFLD is an abbreviation of “nonalcoholic fatty liver disease,” but also that everyone in this community knows that this diagnosis is relevant to the discussion and a reasonable conjecture given the blood test results.

To give a more subtle example, members of our virtual networks mentioned statins and their use frequently in posts and discussions on unrelated topics. Throughout these posts, the role of statins is not immediately evident, as the comments on statins are tangential or incidental to the topic being discussed. Members appeared to embed their knowledge about statins across multiple posts in a nonlinear, intuitive way. For example, in this posting, in a thread about research on gastric bypass (a surgical treatment for obesity), a participant first declares that he is skeptical about the procedure; depicts much of the research on it to be poor quality, conducted by those with vested interests, and heavily influenced by publication bias; and relates some stories of colleagues’ patients who underwent gastric bypass. He then brings in statins as an example of a therapy that is likely to have very limited impact on a problem whose underlying cause lies elsewhere:

I am skeptical about this kind of research, especially as long as they are conducted at private clinics and gastro surgical departments. that promote this. It’s possible I told this story before, but I take my chance to tell it again. I worked on one of the largest hospitals in the laparoscopic pediatric surgery. A colleague was tasked with the making prospective study of laparoscopic vs. conventional appendectomy. The results proved to be so discouraging that all “material” was thrown in the shredder. Unfortunately, the pharmaceutical industry keeps this going. I find it reassuring that X writes that we know too little about long term effects of such operations and that the Knowledge Centre needs to look into this. Recently received input from a few colleagues about bypass surgery. One patient weighing 125 kg before surgery, and now weighs 170 plus. My point is that we need to add significantly more “weight” to preventive health. It is quite pointless to repair, be with it statins, ACB, operations and other means.

Post on Eyr

Here, the reference to statins could be explained by members of the community as fitting in an argument of alternative interventions to reduce cardiovascular risk, but this remains subtle and implicit. The poster—probably rightly—assumes that his colleagues will understand the context in which he is referring to statins. And thus, the poster makes a small contribution to a wider “mindline” on the limited efficacy of statins in the face of multiple risk factors and social determinants.

Another example of tacit knowledge transmission was the use of images and figures. Pictures of parts (a tongue, an eye, and some skin) of unrecognizable, consenting patients were posted in four discussions in our dataset, accompanied with a brief wording, along with tangential mention of the use of statins. Most of these involved diagnosing dermatological cases that are highly dependent on pattern recognition. For example, one person posted a picture of an unusual rash, and (in a thread where several others had offered guesses as to the diagnosis) another GP suggested that this may be a vasculitic rash related to an adverse drug reaction, saying he had seen a very similar rash in such circumstances before. Although the other posters in this thread were attempting to elucidate the diagnosis rationally from particular features of the rash, this poster was relying on intuitive knowledge that he was unable to (and/or did not find it necessary to) articulate in words.

Another important way tacit knowledge was shared in these online forums was through GPs’ postings on what they would do, without citing any formal sources of evidence or explaining their reasoning. On the TGG group, such practical knowledge exchange was common. For example:

Personally, I would choose to reduce alcohol and assess to solve or aid in reducing alcohol while give her a referral for 2 year test.

Post on TGG

Similarly, GPs often suggested what might or might not work, with either no justification or a justification (as in the example below) in which some of the rationale was implicit rather than explicit. In the case below, the poster is arguing that the relatively rare (and usually mild) side effect of depression on statins could become life-threatening in patients with a history of severe depression:

I would not even use statins, without having much harder risk factors than the currently loaded policy. As a psychiatrist, I treated a few patients which were so depressed that they needed hospitalization, shortly after starting with statins. And rhabdomyolysis by individual interactions and other unforeseen circumstances, is simply not to be trifled with.

Post on Eyr

At the same time, these statements often reveal not just how patients should be treated, but also personal experience and internalized preferences of the members themselves. For example, in a discussion on a case where patient has a very high cholesterol level, but (taking other risk factors such as weight and blood pressure into account) a moderate risk of cardiovascular events, members discuss what would be the appropriate thing to do. In commenting on his own post, the initiator of the discussion wrote:

If it was me, I’d want a statin. I think that’s what my gut says.

Post on TGG

In this example, the member is expressing his professional disagreement with imposed policy, referring to how he would feel if he were the patient. In some discussions, members even disclosed their own or their family members’ conditions and how they dealt with them, what medication they would take, and how they experienced the services they used (as patients). For example, in one thread, a member presented the case of a patient with raised levels of cholesterol and a positive family history of high cholesterol, in which she states that this is her husband. She is concerned that his GP only discussed the matter via telephone and wonders whether it would have been appropriate for her husband to go to a specialist lipid clinic. This posting, and the responses to it, illustrate how clinicians’ professional knowledge could not be easily separated from their personal experience of illness and risk. Rather than distant abstract explicit facts, clinical knowledge is embodied and concrete, directly affecting these clinicians themselves.

#### Collective Reinforcement of Knowledge

The online communities served as a collegial fellowship of professional peers where the expected format is for one person to ask for advice and others to share their experiences and reflections. The discussants do not provide final answers, instead, they ask additional questions and give comments, suggestions, or theories that should be subject to further experiments and testing. Each answer is not inherently right or wrong, but the multitude of answers provides a broader sense of what is going on and the options for taking action. This is the means by which a knowledge base gets constructed to support a decision toward multiple actions appropriate for the situation at hand:

A: 17 year old pt has total cholesterol of 7.9 and LDL 5.6. no other risk factors. no family history of heart disease or high lipids, What would you do?

B: Why was it done then!!

B: Diet, weight.

C: Send the blood result to the one that ordered it for them to deal with. She/He should know why they requested it!

D: familial hypercholesterolemia...send to lipid clinic and family too.

E: Bounce straight back to the one who ordered it. They can decide to repeat it instead of basing a lifetime of advice on 1 possibly accidental outcome. Never do a test if (a) you don’t want to know the result and/or (b) will not be able to interpret. Unless you are about to go on vacation or quit that job.

Post on TGG

As a basic part of the concept of mindlines, the knowledge about statins is collectively reinforced through all kinds of social interactions and situations. In most case, the purpose remains largely clinical, but members also wrote about statins and lipids outside the context of clinical practice (eg, in relation to the allegedly unethical behavior of pharmaceutical companies in promoting overdiagnosis and overtreatment). Medical knowledge about statins was embedded in a wider array of stories, jokes, and noteworthy events. For instance, in a TGG group discussion about a cardiologist who advised to replace margarine with butter, a member states that he likes butter for many reasons and posts a picture from an unappetizing cartoon figure ripping his shirt open, revealing his torso, with the words “grease me up woman!”

#### Identity and Social Support

As in other topic threads, knowledge about statins was built and shared in postings that also served to support social cohesion and interaction or describe an atmosphere. For instance, a member started a discussion to express her joy at seeing other members of this virtual group at a face-to-face event. A member, who apparently was not at the face-to-face event, asked:

So, what was the buzz? Was it statins versus stilettos or vests versus venlafaxine? Xxx.

Post on TGG

Other members added to this discussion (talking about cardigans, the need for more gossip, and that they should meet up again face-to-face). GPs showed great affection for the group; throughout the TGG network there were many postings that they enjoyed the interactions and the contact with peers. For instance, one discussion thread began with a member ventilating about an afternoon surgery in which there had been many complex cases. Participants expressed their support in several ways, and then one participant interjected with the following:

Been a regular here for such a long time...I love this group.

Post on TGG

Such socioemotional contributions appeared very important, especially when members were posting about the stressful and challenging aspects of their job. In addition, these kinds of posts show an understanding of knowledge as relational on many levels: as indivisibly linked to the speaker, intrinsically interwoven with other concepts, as a mean to connect with others, and to form and sustain a community.

## Discussion

### Characterizing Online Knowledge and Knowledge Processes

The three virtual networks in this study support knowledge processes in an unstructured, self-organizing way. They are not about translation in a direct sense but intermediation [56]: “the messy engagement of multiple players with diverse sources of knowledge” where a limited set of rules and nudges are imposed by administrators and software. As concrete software tools, these networks can be understood as knowledge artifacts “holding” mindlines but at the same time as “collaboratively created artifacts for knowing” to support the development and sharing of mindlines [40]. This blurred duality of “knowledge” and “knowledge development and processing” encapsulated in one single concept is also key to understand what mindlines contribute to the debate on EBHC. It reminds clinicians, researchers, and guideline developers to stop thinking of knowledge as a simple package, ready to be “implemented” in every day clinical practice. This may be old news in other research fields and traditions (most notably in the social sciences), but not in health care where the often-unquestioned love for “facts” from RCTs require stronger rebuttal. We would contend that our findings inspired by the concept of mindlines provide good arguments to move beyond current evidence-based practice.

First, our findings support the idea of knowledge and knowing as a social practice of a group, consistent with literature on communities of practice and organizational learning (eg, [57,58]). Members of the networks have a shared interest and purpose (providing health care), they interact and learn together (asking questions, contributing to discussions, showing affection, and behaving like a fellowship of colleagues), and they share a repertoire of routines to solve the problems they face (ways of presenting a case, the use of jargon, and critiquing research evidence).

Second, our findings show that on these networks, both explicit and not-so-explicit knowledge is present. Nonexplicit, tacit, and practical knowledge is present on virtual networks, but in different shapes. It can be seen in jargon, images, stories, and “I would do”–statements.

Third, online knowledge on these networks adhered to the concept of mindlines in that it could be characterized as “knowledge-in context-in practice” [17]. In the example of the prescription module, we see how prescription of GPs is accessible to distant others who are monitoring and rewarding or punishing the GPs in ways that put pressure on clinical practice. Such as the Foucauldian panopticon, knowledge and knowledge processes form and are embedded in power relations [59]. By explaining the problems they faced when implementing recommendations, posters offered other members useful insights into the regulations, quality frameworks, and payments schemes in what Larry May has coined a ”web of commitments” [60]. For instance, a GP’s duty is not merely to apply the guideline to a single patient appropriately, but also to maximize health gains from a limited public fund and/or acknowledge the patient’s limited means to pay for the treatment. This could be interpreted as contextual reality (social, financial, legal, and so on) “pushing back” [61] on clinical knowledge and recommendations, complicating them, but also reducing their abstractness and operationalizing the knowledge, making it practical and relevant for the task at hand, right there, right then.

Fourth, storytelling and casuistry are evident in our online datasets. The stories shared tended to be case-oriented, focusing on unusual, rare, or extreme events. This is not a peculiar characteristic of the online environment. On the contrary, it has been well described and analyzed in a philosophical paper addressing the paradox of using extremely rare cases to teach students about more common conditions (“when you hear hoof beats, don’t think zebras” [62]). However, it perhaps illustrates that the virtual network is being used in particular to *extend* clinical knowledge.

We observed a more *telegraphic* format online than the classic narratives shared among clinicians in face-to-face interactions [30,31].

The cases largely lacked the “biographic and social context of the illness experience” [63] present in everyday clinical practice. A hybrid kind of case-based inductive inference occurred: casuistry [32] and pathology-based at the same time, with the main focus on test results and other quasi-quantitative facts about the individual patient (such as whether and how much they smoked). Notwithstanding that this approach may be partly because of the topic (cholesterol as a blood test–based risk factor), the format and style of discussion contrasts markedly with the discussions among GPs in a bygone era in Balint groups, where the central focus of case presentations was the patient’s subjective narrative and the unfolding of the interpersonal GP-patient relationship in an overtly psychodynamic framing [64].

### Validity

Mindlines lack of a theory of validity of knowledge, but based on our findings, we would suggest that validity in online communities appears to be about what works as kind of pragmatic reasoning. If we assess the popularity of some of these networks, we assume that users find much value in them. But the knowledge on these networks almost never clearly comes to a conclusion or single recommendation based on a criterion of correspondence (to reality) or coherence (fitting into a web of beliefs). Rather, their value seems to lie in their ability to support a practical decision in the here and now.

The clinicians in these networks tend to say what they would do or suggest what (in their view) might work. The personal commitment to these suggestions is stressed: “personally...” or “I would say...” In making these suggestions and asking additional questions, it appears they do not mind exploring issues of uncertainty. Indeed, by attending to these uncertainties, they seem to gain a kind of knowledge that is useful for them. Uncertainty itself “is not a regrettable and unavoidable aspect of decision making but a productive component of clinical reasoning” [65].

This relates to Weick’s concept of “collective sensemaking” that is embedded in the theory on mindlines. Through discussion, we make things comprehensible in the best way we can collectively, because an ultimate truth or reality remains uncertain [66]. As the ultimate truth cannot be known, discussants are “scratching around” to make the world as understandable as they can, and only they can judge whether these understandings are useful [66]. As Wittgenstein observed, some problems are readily solved by the addition of data; others (especially those for which a simple answer is impossible) are solved by a *deepening of understanding* [67].

By saying what they would do personally, members appear to adhere to a criterion for valid knowledge similar to pragmatic theories of truth as defined by James: “Ideas...become true just in so far as they help us to get into satisfactory relations with other parts of our experience” [68]. But not fully, as our results show that many views do not come with a clear function and can be contradictory, disjointed, on a tangent, or unclearly related.

An alternative way to look at validity in online virtual networks with a lack of final conclusions would be to use Michail Bakhtins’ concept of polyphonic or unified truth [69]. Bakthin was an early twentieth century Russian philosopher and literary analyst who posed that truth is not construed from one dominant perspective but from the interaction between multiple perspectives from many participants, each with their own validity. He refers to an author of a story who does not present his authoritative truth but lets his or her characters voice many (even contradictory) views that together form the narrative’s “polyphonic” reality. Truth is better conceptualized not as a single thought or post but as the sum total of interactions of posted perspectives, including areas of dissonance and disagreement. This is more than just the summation of those posts; it is an emergent property.

Bakhtin also helps to cope with another problem of mindlines as presented on these online networks. Most network members don’t contribute any posts, only a minority of members do. In how far do the posts represent the *true* mindlines of the collective? This problem is not just limited to mindlines or online networks but to all notions of collective knowledge. In Bakhtin’s view on truth, what is not shared degenerates. Ideas can only thrive and become truthful if they engage in dialogic relationships with other ideas.

### Limitations and Further Research

Although the field of Internet research should not be seen as a new Kuhnian paradigm—an entirely new kind of social science—it is a novel addition to and challenger of older methods to study social relations [70]. As Christine Hine wrote, “Internet research has arguably been a valuable reflexive opportunity for the traditional disciplines that have fed its development” [71]. Reflecting on the novel principles of digital ethnography as suggested by Pink et al [44], this study has several limitations and elicits areas for further research.

Regarding multiplicity, we studied the three networks that each represent different ways in which clinicians can engage with the digital communities: an email-based network, a professional network, and a commercial network. Further research could be helpful to understand these matters better.

As to nondigital-centricness, we have not yet examined how these virtual communities fit into the nondigital lives of their members. Other research would be necessary to do this. However, this study aimed to characterize informal medical knowledge in the medical community to enable further research in how this knowledge relates to guidelines, not how it correlates with actions in medical practice. Further research would be helpful to explore how knowledge of groups in virtual networks relates to informal knowledge of groups of clinicians in other social spaces.

About openness, we experienced an informative interaction especially with the administrators and the members on the TGG group early on in the research. The person collecting the data (SW) was known to the community as a researcher in EBHC as such, but this is unlikely to have led to any significant distortions in the content of the discussion as he did not participate actively in the discussions held. These contributions of the administrators and members greatly helped to shape the study to its current form. However, the HAWeb and Eyr communities were less responsive, possibly because of their lower activity. In our further research, we would aim to find additional means to increase interactivity with members to shape the research.

With regard to reflexivity, we acknowledge the importance to be critical of how the outcomes of this research were produced. Limitations of this study are the narrow scope of the topic and the limited number of virtual networks. Furthermore, the defined time frame may not reflect how the activity (number of posts and people) on the networks changed over time. This makes it impossible to state confidently that the findings reflect online discussions generally rather than discussions on a particular topic. As such, the findings are preliminary. A broader set of topics or other virtual networks may have revealed additional characteristics of knowledge on virtual networks. Further research could aim to pick a contrasting clinical topic such as mental health, and we would welcome other research studies to look into communities of clinicians to describe knowledge processes.

Concerning the principle unorthodoxy, we aim to continue the dialogue with the research participants, also regarding this and future publications on this topic.

### Conclusions

Our findings would be consistent with a definition of web-based clinical knowledge, knowledge creation, and knowledge translation in one single concept as mindlines, seeing them as instruments produced by clinicians to base their decisions on; a lubricant for explicit and tacit knowing shared among social groups and reinforcing norms of good practice in a fluid, dynamic, and constantly evolving way.

The far less structured interactions on the virtual social networks in this research represent a broad understanding of knowledge associated with many important knowledge theories that may be of use for the guideline community. Our findings show that not all networks will provide deep, rich knowledge. But it offers sufficient support to anticipate that analyzing certain virtual social networks as part of guideline update processes could help to better frame and synchronize recommendations with the mindlines of clinicians. It could highlight new topics for guidance updates, find what guidance needs to be formulated better, and evaluate uptake of recommendations. Conversely, this further research should inform whether it is possible—and if so how—to make the links between guidance and clinical practice closer, potentially by using online networks.

## Abbreviations

EBHC: evidence-based health care
EBM: evidence-based medicine
GP: general practitioner
NAFLD: Nonalcoholic fatty liver disease
NHG: Nederlands Huisarts Genootschap (Dutch College of General Practitioners)
NICE: National Institute for Health and Care Excellence
RCT: randomized controlled trial
TGG: Tiko’s GP Group
